# Eastes v. Russ

**Published:** 1913-05-31

**Authors:** 


### Eastes v. Russ.

MR. JUSTICE SARGANT'S JUDGMENT.

As we go to press, Mr. Justice Sargant's judgment in the case of Eastes *v.* Russ is available. The plaintiff, Mr. G. L. Eastes, M.B., of 38 Cavendish Street, brought an action to restrain Mr. Charles Russ, M.B., of 25 Beaumont Street, from carrying on within ten miles from Queen Anne Street laboratory work similar to that which he had performed when employed by Mr. Eastes. The plaintiff contended that Clause 12 in the terms of engagement bound the defendant in this respect. Among the points to be decided was whether on a true construction of this clause the obligation was only to continue during the term of the employment, or for the whole of the defendant's life. The action was dismissed, with costs. As, however, the judgment raises several points of interest to the medical profession, we hope to refer to it in greater detail next week.

